# Frequency-Dependent Changes in the Regional Amplitude and Synchronization of Resting-State Functional MRI in Stroke

**DOI:** 10.1371/journal.pone.0123850

**Published:** 2015-04-17

**Authors:** Jianfang Zhu, Yuanyuan Jin, Kai Wang, Yumiao Zhou, Yue Feng, Maihong Yu, Xiaoqing Jin

**Affiliations:** 1 Department of Acupuncture and Moxibustion, Zhejiang Hospital, Number 12, Lingyin Road, Xihu District, Hangzhou, China; 2 Department of Neurology, Zhejiang Hospital, Number 12, Lingyin Road, Xihu District, Hangzhou, China; 3 Department of Radiology, Zhejiang Hospital, Number 12, Lingyin Road, Xihu District, Hangzhou, China; Zhejiang Key Laboratory for Research in Assesment of Cognitive Impairments, CHINA

## Abstract

Resting-state functional magnetic resonance imaging (R-fMRI) has been intensively used to assess alterations of inter-regional functional connectivity in patients with stroke, but the regional properties of brain activity in stroke have not yet been fully investigated. Additionally, no study has examined a frequency effect on such regional properties in stroke patients, although this effect has been shown to play important roles in both normal brain functioning and functional abnormalities. Here we utilized R-fMRI to measure the amplitude of low-frequency fluctuations (ALFF) and regional homogeneity (ReHo), two major methods for characterizing the regional properties of R-fMRI, in three different frequency bands (slow-5: 0.01-0.027 Hz; slow-4: 0.027-0.73 Hz; and typical band: 0.01-0.1 Hz) in 19 stroke patients and 15 healthy controls. Both the ALFF and ReHo analyses revealed changes in brain activity in a number of brain regions, particularly the parietal cortex, in stroke patients compared with healthy controls. Remarkably, the regions with changed activity as detected by the slow-5 band data were more extensive, and this finding was true for both the ALFF and ReHo analyses. These results not only confirm previous studies showing abnormality in the parietal cortex in patients with stroke, but also suggest that R-fMRI studies of stroke should take frequency effects into account when measuring intrinsic brain activity.

## Introduction

Mapping the post-stroke brain alterations is of great clinical interest in stroke study as it can help evaluate the post-stroke brain deficits or monitor the rehabilitation effects. Functional MRI (fMRI) has been increasingly used to assess the functional alterations in stroke.

fMRI is a powerful and non-invasive tool to delineate functional abnormality in the brain after stroke [1–4]. Earlier studies have mainly used task-based fMRI and identified abnormal local activities and inter-regional connectivity involving the fronto-parietal motor control systems [5], the sensorimotor cortex [6–7], inferior parietal lobule region and cerebellum regions[8], temporo-parietal area [7], fronto-temporo-occipital area, supramarginal gyrus, angular gyrus, and basal ganglia and fusiform gyrus [9]. However, task-based fMRI in stroke studies often suffers from a large variability due to the difficulty of patients experience in performing the tasks [10]. By contrast, resting-state fMRI (R-fMRI) does not require participants to perform any explicit tasks, and therefore is particularly suitable for this specific population [11]. R-fMRI measures spontaneous brain activity as low-frequency fluctuations in blood oxygen level-dependent (BOLD) signals. These low-frequency fluctuations are vital for a better understanding of human brain function under both healthy and pathological conditions because extremely disproportionate energy consumption appears within the regions showing high resting metabolic rates [12–14]. Indeed, the number of R-fMRI studies in stroke has dramatically increased in the recent years. Notably, the majority of R-fMRI studies in stroke have focused on the characterization of inter-regional functional connectivity, such as connections between the motor systems and non-motor systems [15–16]. While regional properties are important to understand the neural pathology of stroke and its recovery, only one study has examined such regional properties in patients with subcortical stroke [17]. The present study aimed to address this gap.

Amplitude of low-frequency fluctuations (ALFF) and regional homogeneity (ReHo) are two widely used approaches that have high test-retest reliability [18–19] in characterizing the local brain features of R-fMRI data. Specifically, the ALFF quantifies the strength or intensity of low-frequency (typically 0.01–0.1 Hz) oscillations (LFOs) embedded in the spontaneous neural activity while the ReHo reflects the statistical similarity of spontaneous neural activity, among spatially adjacent brain tissues [19]. These two methods have been widely used to explore local functional abnormalities in a variety of brain disorders [21–24]. Of particular importance, recent studies indicate that different frequency bands contribute differently to the LFOs [20, 25–26] and frequency-dependent changes in LFOs have been reported in various brain disorders [27–28]. Therefore, in the current study, we directly tested whether stroke-related changes in local brain regions are also frequency dependent.

We recruited 19 patients with stroke and 15 age- and gender-matched healthy controls, and each paricipant underwent a R-fMRI scanning. We then calculated individual ALFF and ReHo maps in the following three frequency ranges: 0.01–0.1 Hz, which is typically used in the R-fMRI community (termed as the full frequency band); 0.01–0.027 Hz (termed as the slow-5 frequency band); and 0.027–0.073 (termed as the slow-4 frequency-band). Finally, cross sectional ALFF and ReHo differences between the stroke patients and healthy controls were then assessed using two-sample t-tests.

## Materials and Methods

### Participants

Nineteen patients with infarcts in the middle cerebral artery (MAC), diagnosed with computed tomography angiography (CTA) or magnetic resonance angiography (MRA), were selected at Zhejiang Hospital, and 15 age- and gender-matched healthy controls were included in this study. The inclusion criteria were as follows: (1) first occurrence of stroke, and the time poststroke was 2 weeks-1 months; (2) right-handedness; (3) normal cognitive functions (Mini-Mental Status Examination [MMSE]>24); (4) hemiparesis with myodynamia ≥ 3 and/or aphasia; and(5) 50–75 years old. The exclusion criteria were as follows: (1) contraindication for MRI; (2) quadriplegia; (3) prior history of neurological or psychiatric disorders; (4) diabetes; and (5) severe aphasia, neglect, or sensory disturbances. There were no significant differences in age (t_26_ = 0.652, p = 0.520) or gender (*χ*_1_ = 1.44, p = 0.705) between stroke patients and normal controls. The clinical and demographic data of the remaining patients are summarized in Table 1.

**Table 1:** Clinical and demographic data of 15 patients with stroke enrolled in this study.

Case	Gender	Age (years)	Lesion	Time poststroke (weeks)
1	F	63	FC.L,MCA.B	2
2	M	62	BG.L	2
3	F	74	BG.L	2
4	M	63	T-PC.R, MCA.R	2
5	M	63	BG.R, CR.R, Ins.R	2
6	M	76	T-OC.L, TC.L	2.5
7	M	62	BG.L	2.5
8	F	72	pons.L,BG.B,IC.B, Put.B, Tha.B	3
9	F	81	IC.L, Put.L, Tha.L	3
10	F	59	BG.L,FC.L	3
11	M	69	BG.L	4
12	M	78	BG.L, Tha.L, CR.L	4
13	F	80	IC.L, Put.L, Tha.L, BG.L	4
14	F	75	CR.R	4
15	F	78	F-PC.L,TS.BG	4

Note: M, male; F, female; L, left; R, right; B, bilateral; BG, basal ganglia; IC, internal capsule; Put, putamen; Ins, insula; Tha, thalamus; CR, coronal radiate; T-PC, temporo-parietal cortex; MCA, middle cerebral artery; T-OC, temporo-occipital cortex; TC, temporal cortex; F-PC, fronto-parietal cortex; PC, parietal cortex; FC, frontal cortex.

This study was approved by the Research Ethics Review Board of Zhejiang Hospital. The study procedures were explained to all of the participants, and written informed consent was obtained from each participant or their guardians/spouses.

### Image Acquisition

All of the participants were scanned using a 3.0T MR scanner (Philips Achieva Magnetom Avanto, Amsterdam Netherlands) at Zhejiang Hospital. T2* weighted R-fMRI data were acquired using an echo-planar imaging pulse sequence: 33 axial slices; repetition time (TR) = 2000 ms; echo time (TE) = 30 ms; slice thickness = 3.5 mm; gap = 0.7 mm; flip angle (FA) = 90°; matrix = 64×64; field of view (FOV) = 200 × 200 mm^2^. During data acquisition, the participants were asked to lie quietly in the scanner with their eyes closed. A total of 240 image volumes were obtained for each participant.

### Data Processing

R-fMRI data preprocessing was performed with the Graph-theoretical Network Analysis (GRETNA) toolbox (http://www.nitrc.org/projects/gretna/) based on the Statistical Parametric Mapping (SPM) 8 software (http://www.fil.ion.ucl.ac.uk/spm/software/spm8/). After removal of the first 5 volumes, the functional images were first corrected for time offsets between slices and geometrical displacements due to head movement. Four participants were excluded because of excessive head movement. The corrected functional data were then normalized to the Montreal Neurological Institute space using an optimum 12-parameter affine transformation and non-linear deformations and then resampled to a 3-mm isotropic resolution. The resulting images further underwent spatial smoothing (Gaussian kernel with a full width at half maximum = 6 mm), linear detrending, and temporal filtering (0.01–0.1 Hz). Such a typical frequency-band filtering ensured the comparability of our results with results in the existing literature.

To examine the frequency effects on brain activity changes in patients, temporal band-filtering was further performed in the slow-5 (0.01–0.027 Hz) and slow-4 (0.027–0.073 Hz) bands, respectively. Finally, several nuisance signals were regressed out from each voxel’s time course, including 24-parameter head-motion profiles (6 head motion parameters, 6 head-motion parameters one time point before and after their corresponding 12 squared items) [29–30], mean white matter (WM), and the cerebrospinal fluid (CSF) time series derived within prior probability maps in SPM8 (threshold = 0.8).

### ALFF/ReHo calculation

#### ALFF

For a given voxel, the time series was first converted to the frequency domain using a Fast Fourier Transform. The square root of the power spectrum was then computed and summed across a predefined frequency interval. This summed square root was the ALFF for this voxel.

#### ReHo

For a given voxel, Kendall’s coefficient of concordance (KCC) [31] was computed on the ranked BOLD time series within a cluster formed by the given voxel and its nearest neighbors (27 voxels in the current study). The resultant KCC was the ReHo for the voxel. Note, spatial smoothing was conducted for individual ReHo maps rather than during data preprocessing to avoid overestimating ReHo values.

All of the ALFF/ReHo computations were restricted in a brain mask where two criteria were fulfilled: (1) grey matter (GM) probability > 0.2 based on a prior GM probability map in SPM8; and (2) non-zero variance over time across all participants.

### Statistical analysis

Two-sample t-tests were used to determine the between-group differences in the ALFF/ReHo data. Given possible residual head motion effects, head-motion variables (maximum, root mean square and mean frame-wise displacement) were treated as non-interest covariates during between-group comparisons [32]. All of the results are presented at a statistical threshold of p < 0.05 (corrected) by combining a height threshold (p < 0.001) and an extent threshold (p < 0.05) determined by Monte Carlo simulations [33].

## Results

### ALFF

**Fig 1:**
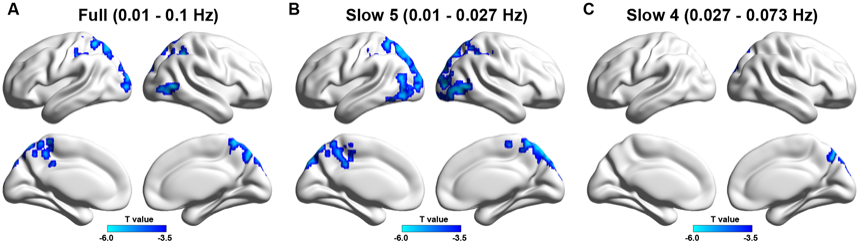
ALFF differences in three different frequency bands (A, typical band: 0.01–0.1 Hz; B, slow-5: 0.01–0.027 Hz; C, slow-4: 0.027–0.73 Hz) between stroke patients and healthy controls. Cold colors indicate regions showing lower ALFF in patients versus the controls. Threshold for ALFF: p< 0.05 (corrected). Left in the figure shows the left side of the brain.

In the full frequency band (0.01–0.1 Hz), 7 clusters were detected that showed decreased ALFF in the stroke patients compared with healthy controls, including the following: right middle temporal gyrus and middle occipital gyrus; right precuneus and superior parietal lobule; right inferior parietal lobule and angular gurus; left superior parietal lobule and precuneus; left inferior and superior parietal lobule; left middle and superior occipital gyrus; and left middle cingulate gyrus and precuneus (p < 0.05, corrected, Table 2, Fig 1A). The between-group differences in the slow-5 band (0.01–0.027 Hz) highly resembled the differences in the 0.01–0.1 Hz band but the voxels with decreased ALFF were spatially more extensive (p < 0.05, corrected, Table 2, Fig 1B).

**Table 2:** Regions showing decreased ALFF in stroke patients versus normal controls.

Brain regions	Hemisphere	CS (voxels)	Peak MNI	Maximum T
Full (0.01–0.1 Hz)				
MTG/MOG	R	122	51,-57,3	6.14
MOG/SOG	L	98	-21,-96,15	5.31
PCu/SPL	R	267	6,-75,42	6.67
IPL/ANG	R	102	36,-48,45	6.22
MCG/PCu	L	89	-6,-42,54	6.03
IPL/SPL	L	121	-33,-54,-60	5.31
SPL/PCu	L	131	-15,-60,51	5.05
Slow 5 (0.01–0.027 Hz)				
ITG/MOG	L	135	-60,-60,-21	5.02
MTG/MOG/IOG	R	206	54,-63,3	5.92
SPL/PCu/MOG/SOG	B	1206	18,-81,45	6.58
Slow 4 (0.027–0.073 Hz)				
PCu/SPL	R	159	6,-75,42	7.22

Note: MTG, middle temporal gyrus; MOG, middle occipital gyrus; SOG, superior occipital gyrus; PCu, precuneus; SPL, superior parietal lobule; IPL, inferior parietal lobule; ANG, angular gurus; MCG, middle cingulate gyrus; ITG, inferior temporal gyrus; IOG, inferior occipital gyrus; L, left; R, right; B, bilateral; CS, cluster size.

However, in the slow-4 band (0.027–0.073 Hz), only the right precuneus and superior parietal gyrus showed decreased ALFF in the patients (p < 0.05, corrected, Table 2, Fig 1C).

These findings suggest that the slow-5 band was primarily responsible for the observed between-group difference in the 0.01–0.1 Hz band, pointing to the sensitivity of the slow-5 band in detecting stroke-related abnormalities of in LFOs.

### ReHo

**Fig 2:**
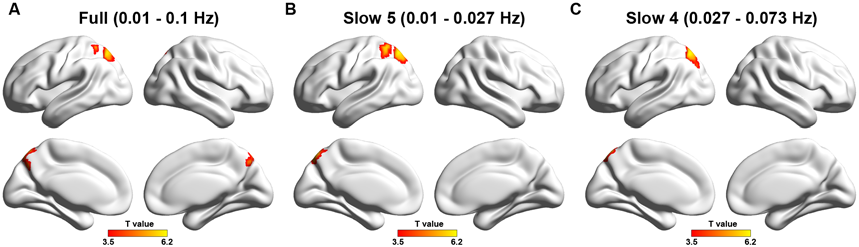
ReHo differences in three different frequency bands (A, typical band: 0.01–0.1 Hz; B, slow-5: 0.01–0.027 Hz; C, slow-4: 0.027–0.73 Hz) between stroke patients and healthy controls. Warm colors indicate regions showing higher ReHo in patients versus controls. Threshold for ReHo: p < 0.05 (corrected). Left in the figure shows the left side of the brain.

In the full frequency band of 0.01–0.1 Hz, a cluster including the left superior parietal lobule and precuneus was identified that showed significantly increased ReHo in the patients in compared with healthy controls (p < 0.05, corrected, Table 3, Fig 2A). In the slow-5 frequency-band (0.01–0.027 Hz), a larger cluster that exhibited decreased ReHo in the patients that was largely overlapped with the cluster detected in the full frequency-band (p < 0.05, corrected, Table 3, Fig 2B). As for the slow-4 frequency-band (0.027–0.073 Hz), although the same cluster was also detected showing increased ReHo in the patients, the spatial extent was reduced by approximately half relative to the extent in the full-frequency-band (p < 0.05, corrected, Table 3, Fig 2C).

**Table 3:** Regions showing increased ReHo in stroke patients versus normal controls.

Brain regions	Hemisphere	CS (voxels)	Peak MNI	Maximum T
Full (0.01–0.1 Hz)				
SPL/PCu	L	270	6,-75,42	6.2
Slow 5 (0.01–0.027 Hz)				
SPL/IPL	L	356	-27,-51,51	6.96
Slow 4 (0.027–0.073 Hz)				
SPL/PCu	L	139	-18,-69,54	5.96

Note: SPL, superior parietal lobule; PCu, precuneus; IPL, inferior parietal lobule; L, left; R, right hemisphere; CS, cluster size.

### Relationship between ALFF and ReHo

**Fig 3:**
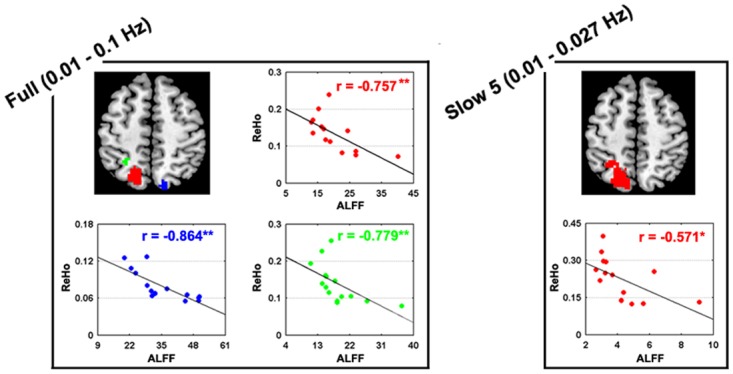
Relationships between ALFF and ReHo for regions showing abnormal ALFF and ReHo in patients. Significantly negative correlations were found between these two measures in stroke patients.

We found that several regions simultaneously exhibited abnormalities of decreased ALFF and increased ReHo in patients compared with healthy controls. Thus, we further examined the relationships between ALFF and ReHo for these regions in the patients. As shown in Fig 3, significant negative correlations were found between these two measures in stroke patients.

## Discussion

In the present study, we examined regional changes in LFOs (i.e., ALFF and ReHo) of R-fMRI signals at different frequency bands in patients with stroke. To our knowledge, there is no prior research using frequency approaches to detect stroke-related R-fMRI changes in the human brain. Using ALFF, we found that patients with stroke showed lower LFO amplitudes compared with controls for the full frequency band (0.01–0.1 Hz) in the right middle temporal gyrus, middle occipital gyrus, precuneus and superior and inferior parietal lobules, angular gurus, and the left precuneus, superior and inferior parietal lobules, and middle cingulate gyrus. The between-group differences in slow-5 band highly resembled the differences in the 0.01–0.1 Hz band, but the voxels with decreased ALFF were spatially more extensive. In contrast, the ALFF in the slow-4 band analysis revealed only one cluster in the right precuneus/superior parietal lobules. Using ReHo, we observed increased synchronization of LFOs in the slow-5 band (0.01–0.027 Hz) in the left inferior/superior parietal lobule in stroke patients compared with normal controls, while within both the full frequency band (0.01–0.1 Hz) and the slow-4 band (0.027–0.073 Hz), we observed increased synchronization in the left superior parietal lobule and precuneus. We next discuss the theoretical and clinical implications of these findings.

### Changed activity in the parietal cortex

The most robust result of the present study is altered brain activity in the parietal cortex of the patients, which was observed when the data were analyzed with both the ALFF and ReHo methods in all three frequency domains. This finding is in accordance with previous R-fMRI studies showing altered inter-regional functional connectivity between the parietal lobule and other brain regions in stroke [15–16]. A recent study, using the ReHo method to study 24 patients with subcortical stroke also documented increased parietal activity in stroke [34]. Therefore, our current study and previous studies by others converge to show abnormality in the parietal cortex. This finding likely has clinical significance. For example, the parietal cortex could serve as a target region for specific rehabilitation therapies, such as transcranial magnetic stimulation.

It should be noted, however, that the mechanisms underlying the changes of parietal activity are currently poorly understood. The present study showed increased ReHo, which is consistent with the results reported by Yin et al. [17], but decreased ALFF in the parietal cortex in patients with stroke. Further analaysis revealed significantly negative correlations between ALFF and ReHo in the parietal regions that simultaneously showed stroke-related abnormalities. Therefore, we speculate that the increased ReHo may be due to the stroke-induced homodromous alterations in spontaneous brain activity (i.e., decreased ALFF), which results in enhanced synchronization. Further, task-based fMRI studies typically show decreased parietal activation in stroke [6–7]. Our current data are not sufficient to interpret these apparent discrepancies, although there are several possibilities. For example, the discrepancies may be attributable to methodological factors such as the use of different analytic methods and tasks, scanning parameters, or even preprocessing procedures and choice of frequency filtering choices. Another possibility is that there is heterogeneity in the clinical phenotypes of stroke across different studies; studies with a larger sample and grouping designs could test this possibility. Therefore, comprehensive studies with a larger sample and using multi-mode MRI are needed to clarify the above discrepancies and deepen our understanding of the neural mechanisms underlying the changed activity in the parietal cortex that have been consistently revealed by the present study and the results of others.

### Frequency dependent effects of R-fMRI

The most important finding of our study is that the abnormal spontaneous neural activity measured by R-fMRI in stroke is frequency dependent. Specifically, changes in LFOs in the slow-5 band were identified in widespread cortical regions, including the parietal lobules, occipital gyrus, precuneus, and temporal gyrus, whereas LFOs in the slow-4 band only showed marginally significant changes that were limited to the parietal lobules. These findings are consistent with earlier findings that LFOs in the slow-5 band showed higher power distributed in widespread cortical regions [21,33], while greater ALFF in the slow-4 band was mainly located in subcortical regions (more robustly in the basal ganglia) [20,25,27,35]. One possible reason for the differential distributions of these two frequency bands of LFOs might be that LFOs are important for the integration of large-scale neuronal networks, whereas higher frequency oscillations are responsible for neuronal communications in a small space [36].

Our finding of frequency-dependent effects has both theoretical and clinical implications. There are other regional brain activity measures such as entropy and variability [37–39] that demonstrate that intrinsic, coherent neuronal signaling may be essential to the development and maintenance of brain functions. Thus, neural oscillations might be the fundamental mechanism not only for coordinated brain activity during normal functioning but also for recovery from brain injury such as stroke and traumatic brain injury.

Our study has several limitations. First, although we instructed participants to close their eyes using an eye mask during scanning, without electroencephalogram (EEG) tracking, we cannot completely rule out the possibility that some of the participants failed to comply with this instruction throughout the resting-state scanning [15]. Second, we only tested a relatively small sample size in each group. Further studies are needed to reproduce our findings by recruiting a large cohort of patients. Finally, although we took great care to minimize participants’ head motion, it is still possible that a small proportion of the effects reported here could be accounted for by motion-induced artifacts. Nevertheless, the consistent findings of changed regions, particularly the parietal cortex, in stroke patients across studies using task-based fMRI or R-fMRI (including our current study), at least partially verify the validity of the results of this study.

In conclusion, the current study demonstrated abnormal LFOs in the parietal cortex, precuneus, temporal gyrus, and occipital gyrus in stroke patients, with the altered activity in the parietal cortex being the most robust finding (consistently identified by all of the methods we used). Importantly, we showed that the abnormalities in LFOs in stroke were frequency dependent. We suggest that R-fMRI studies in stroke and other brain disorders should consider frequency effects.

## References

[1] Smits M, Visch-Brink EG, van de Sandt-Koenderman ME, van der Lugt A. Advanced Magnetic Resonance Neuroimaging of Language Function Recovery After Aphasic Stroke: A Technical Review. Arch Phys Med Rehabil. 2012; 93: 4–14. 10.1016/j.apmr.2011.02.023 22202190

[2] Van Oers CA, Vink M, van Zandvoort MJ, van der Worp HB, de Haan EH, kappelle LJ, et al Contribution of the left and right inferior frontal gyrus in recovery from aphasia. A functional MRI study in stroke patients with preserved hemodynamic responsiveness. NeuroImage. 2010; 49: 885–893. 10.1016/j.neuroimage.2009.08.057 19733673

[3] Anderson, Ferguson, Lopez-Larson, Yurgelun-Todd. Reproducibility of single-subject functional connectivity measurements. American journal of neuroradiology. 2011; 32(3): 548–55. 10.3174/ajnr.A2330 21273356 PMC3205089

[4] Van Dijk KR, Hedden T, Venkataraman A, Evans KC, Lazar SW, Buckner RL. Intrinsic functional connectivity as a tool for human connectomics: theory, properties, and optimization. J Neurophysiol. 2010; 103(1): 297–321. 10.1152/jn.00783.2009 19889849 PMC2807224

[5] Inman CS, James GA, Hamann S, Rajendra JK, Pagnoni G, Butler AJ. Altered resting-state effective connectivity of fronto-parietal motor control systems on the primary motor network following stroke. NeuroImage. 2012; 59: 227–237. 10.1016/j.neuroimage.2011.07.083 21839174 PMC3195990

[6] Dechaumont-Palacin, Marque, De Boissezon, Castel-Lacanal, Carel, Berry, et al Neural Correlates of Proprioceptive Integration in the Contralesional Hemisphere of Very Impaired Patients Shortly After a Subcortical Stroke: An fMRI Study. Neurorehabil Neural Repair. 2008; 22: 154–165. 17916656 10.1177/1545968307307118

[7] Jung TD, Kim JH, Seo JH, Jin SU, Lee HJ, Lee SH, et al Combined information from resting-state functional connectivity and passive movements with functional magnetic resonance imaging differentiate fast late-onset motor recovery from progressive recovery in hemiplegic stroke patients: a pilot study. J Rehabil Med. 2013; 45: 546–552. 10.2340/16501977-1165 23695814

[8] Cho SY, Kim M, Sun JJ, Jahng GH, Kim HJ, Park SU, et al A Comparison of Brain Activity between Healthy Subjects and Stroke Patients on fMRI by Acupuncture Stimulation. Chin J Integr Med. 2013; 19: 269–276. 10.1007/s11655-013-1436-4 23546630

[9] van Oers CA, Vink M, van Zandvoort MJ, van der Worp HB, de Haan EH, Kappelle Lj, et al Contribution of the left and right inferior frontal gyrus in recovery from aphasia: A functional MRI study in stroke patients with preserved hemodynamic responsiveness. NeuroImage. 2010; 49: 885–893. 10.1016/j.neuroimage.2009.08.057 19733673

[10] Daly JJ, Hrovat K, Pundik S, Sunshine J, Yue G. fMRI methods for proximal upper limb joint motor testing and identification of undesired mirror movement after stroke. Journal of Neuroscience Methods. 2008; 175: 133–142. 10.1016/j.jneumeth.2008.07.025 18786565

[11] Shehzad Z, Kelly AM, Reiss PT, Gee DG, Gotimer K, Uddin LQ, et al The resting brain: unconstrained yet reliable. Cereb Cortex. 2009; 19(10): 2209–2229. 10.1093/cercor/bhn256 19221144 PMC3896030

[12] Raichle ME. The brain's dark energy. Science. 2006; 314: 1249–1250. 17124311

[13] Raichle ME, MacLeod AM, Snyder AZ, Powers WJ, Gusnard DA, Shulman GL. A default mode of brain function. Proc Natl Acad Sci USA. 2001; 98: 676–682. 11209064 10.1073/pnas.98.2.676 PMC14647

[14] Fox MD, Raichle ME. Spontaneous fluctuations in brain activity observed with functional magnetic resonance imaging. Nat Rev Neurosci. 2007; 8: 700–711. 17704812 10.1038/nrn2201

[15] Várkuti B, Guan C, Pan Y, Phua KS, Ang KK, Kuah CW, et al Resting State Changes in Functional Connectivity Correlate With Movement Recovery for BCI and Robot-Assisted Upper-Extremity Training After Stroke. Neurorehabilitation Neural Repair. 2013; 27: 53–62. 10.1177/1545968312445910 22645108

[16] Golestani AM, Tymchuk S, Demchuk A, Goodyear BG. Longitudinal Evaluation of Resting-State fMRI After Acute Stroke With Hemiparesis. Neurorehabil Neural Repair. 2013; 27: 153–163. 10.1177/1545968312457827 22995440

[17] Yin D, Luo Y, Song F, Xu D, Peterson BS, Sun L, et al Functional reorganization associated with outcome in hand function after stroke revealed by regional homogeneity. Neuroradiology. 2013; 17: 19–22. 10.1186/1687-9856-2013-17 23417103

[18] Zuo XN, Di Martino A, Kelly C, Shehzad ZE, Gee DG, Klein DF, et al The oscillating brain: Complex and reliable. Neuroimage. 2010; 49: 1432–1445. 10.1016/j.neuroimage.2009.09.037 19782143 PMC2856476

[19] Li Z, Kadivar A, Pluta J, Duniop J, Wang Z. Test-retest stability analysis of resting brain activity revealed by blood oxygen level-dependent functional MRI. Journal of magnetic resonance imaging. 2012; 36: 344–354. 10.1002/jmri.23670 22535702 PMC3399952

[20] Zuo XN, Di Martino A, Kelly C, Shehzad ZE, Gee DG, Klein DF, et al The oscillating brain: Complex and reliable. NeuroImage. 2010; 49: 1432–1445. 10.1016/j.neuroimage.2009.09.037 19782143 PMC2856476

[21] He Y, Wang L, Zang Y, Tian L, Zhang X, Li X, et al Regional coherence changes in the early stages of alzheimer’s disease: a combined structural and resting-state functional MRI study. Neuroimage. 2007; 35: 488–500. 17254803 10.1016/j.neuroimage.2006.11.042

[22] Wang Z, Yan C, Zhao C, Qi Z, Zhou W, Lu J, et al Spatial patterns of intrinsic brain activity in mild cognitive impairment and Alzheimer's disease: a resting-state functional MRI study. Hum Brain Mapp. 2011; 32: 1720–1740. 10.1002/hbm.21140 21077137 PMC6870362

[23] Zang YF, He Y, Zhu CZ, Cao QJ, Sui MQ, Liang M, et al Altered baseline brain activity in children with ADHD revealed by resting-state functional MRI. Brain Dev. 2007; 29: 83–91. 16919409 10.1016/j.braindev.2006.07.002

[24] Hoptman MJ, Zuo XN, Butler PD, Javitt DC, Angelo D, Mauro C, et al Amplitude of low-frequency oscillations in schizophrenia: a resting state fMRI study.Schizophr. Res. 2009; 117: 13–20. 10.1016/j.schres.2009.09.030 19854028 PMC2822110

[25] Baria AT, Baliki MN, Parrish T, Apkarian AV. Anatomical and functional assemblies of brain BOLD oscillations. J Neurosci. 2011; 31:7910–7919. 10.1523/JNEUROSCI.1296-11.2011 21613505 PMC3114444

[26] Han Y, Lui S, Kuang W, Lang Q, Zou L, Jia J. Anatomical and functional deficits in patients with amnestic mild cognitive impairment. PLoS one. 2012; 7: e28664 10.1371/journal.pone.0028664 22319555 PMC3272002

[27] Han Y, Wang J, Zhao Z, Min B, Lu J, Li K, et al Frequency-dependent changes in the amplitude of low-frequency fluctuations in amnestic mild cognitive impairment: A restingstate fMRI study. NeuroImage. 2010; 55: 287–295 10.1016/j.neuroimage.2010.11.059 21118724

[28] Di Martino A, Ghaffari M, Curchack J, Reiss P, Hyde C, Vannucci M, et al Decomposing intra-subject variability in children with attention-deficit/hyperactivity disorder. Biol Psychiatry. 2008; 64: 607–614. 10.1016/j.biopsych.2008.03.008 18423424 PMC2707839

[29] Friston KJ, Wiliams S, Howard R, Frackowik RS, Tumer R. Movement-related effects in fMRI time-series. Magn Reson Med. 1996; 35: 346–355. 8699946 10.1002/mrm.1910350312

[30] Yan CG, Cheung B, Kelly C, Colcombe S, Craddock RC, Di Martino A, et al A comprehensive assessment of regional variation in the impact of head micromovements on functional connectomics. Neuroimage. 2013; 76: 183–201. 10.1016/j.neuroimage.2013.03.004 23499792 PMC3896129

[31] Nunn, Smith. Statistical analyses of developmental sequences: the craniofacial region in marsupial and placental mammals. Am Nat. 1998;152: 82–101. 10.1086/286151 18811403

[32] Fair DA, Niqq JT, lyer S, Bathula D, Mills KL, Dosenbach NU, et al Distinct neural signatures detected for ADHD subtypes after controlling for micro-movements in resting state functional connectivity MRI data. Front Syst Neurosci. 2012; 6: 80–87. 10.3389/fnsys.2012.00080 23382713 PMC3563110

[33] Ledberg A, Akerman S, Roland PE. Estimation of the probabilities of 3D clusters in functional brain images. Neuroimage. 1998; 8: 113–128. 9740755 10.1006/nimg.1998.0336

[34] Dazhi Y, Yanli L, Fan S, Dongrong X, Peterson BS, Sun L, et al Functional reorganization associated with outcome in hand function after stroke revealed by regional homogeneity. Neuroradiology. 2013; 17: 19–22. 10.1186/1687-9856-2013-17 23417103

[35] Rongjun Y, Yi-Ling C, Hsiao-Lan SW, Liu CM, Liu CC, Hwang Tj, et al Frequency-Specific Alternations in the Amplitude of Low-Frequency Fluctuations in Schizophrenia. Human Brain Mapping. 2012; 0000: 00–12. 10.1002/hbm.22203 PMC6869729 23125131

[36] Buzsaki G, Draguhn A. Neuronal oscillations in cortical networks. Science. 2004; 304: 1926–1929. 15218136 10.1126/science.1099745

[37] Altamura C, Reinhard M, Magnus-Sebastian V, Kaller CP, Hamzei F, Vernieri F, et al The longitudinal changes of BOLD response and cerebral hemodynamics from acute to subacute stroke. A fMRI and TCD study. Neuroscience. 2009; 10: 151 10.1186/1471-2202-10-151 20021696 PMC2805667

[38] Wang Z, Li Y, Childress AR, Detre JA. Brain entropy mapping using fMRI, Plos One. 2014; 9(3): e89948 10.1371/journal.pone.0089948 24657999 PMC3962327

[39] Garrett DD, Kovacevic N, Mclntosh AR, Grady CL. Blood oxygen level-dependent signal variability is more than just noise. J Neurosci. 2010; 30(14):4914–21. 10.1523/JNEUROSCI.5166-09.2010 20371811 PMC6632804

